# Effectiveness of Traditional Chinese Medicine for Liver Protection and Chemotherapy Completion among Cancer Patients

**DOI:** 10.1093/ecam/nep185

**Published:** 2011-06-07

**Authors:** Mei-Ling Liu, Li-Yin Chien, Cheng-Jeng Tai, Kuan-Chia Lin, Chen-Jei Tai

**Affiliations:** ^1^Department of Nursing, Taipei Veterans General Hospital, Taiwan; ^2^Institute of Clinical and Community Health Nursing, National Yang-Ming University, Taiwan; ^3^Department of Internal Medicine, Taipei Medical University Hospital, Taiwan; ^4^Department of Medicine, Taipei Medical University, Taiwan; ^5^School of Nursing, National Taipei College of Nursing, Taiwan; ^6^Department of Traditional Chinese Medicine, Taipei Medical University Hospital, Taiwan; ^7^Department of Medicine, Taipei Medical University, Taipei, Taiwan

## Abstract

While traditional Chinese medicine (TCM) is widely used among Chinese patients with cancer, studies evaluating the effectiveness of TCM using objective indicators are rare. We examined the effectiveness of TCM for liver protection and completion of chemotherapy among patients with cancer receiving chemotherapy. We used a case-control design to examine the medical records of patients with cancer who received chemotherapy in a teaching hospital in Taipei in 2004. A total of 184 courses of chemotherapy among 89 patients were studied. Of the 184 courses, 42 used TCM jointly with chemotherapy served as cases, while the remaining 142 courses served as controls. Outcome variables included counts of cancelled or delayed chemotherapies and liver function (aspartate aminotransferase, AST and alanine aminotransferase, ALT) 1 week before, during and 2 weeks after chemotherapy. Generalized estimating equations were used to analyze the data. Patients who had concomitant TCM with chemotherapy had lower serum ALT and AST during chemotherapy than the controls given that the age, sex, cancer stage, radiotherapy sites, cancer diagnosis and potential hepatotoxicity of the chemotherapeutic drugs were controlled for in the model [**β**  = −3.48, 95% confidence interval (CI) −10.08 to 3.11 for AST; **β**  = −5.95, 95% CI: −11.47 to −0.44 for ALT]. There was no significant difference between the case and control groups for odds of completing one course of chemotherapy. Use of TCM with chemotherapy resulted in protection of the liver during chemotherapy, as manifested by lower serum AST and ALT levels.

## 1. Introduction

Hepatotoxicity is a common side effect of chemotherapy [1]. Prevalence of hepatotoxicity ranges from 33 to 65.6% among patients with cancer [2, 3], and up to 30% of patients have the third or fourth degree hepatotoxicity [4]. Although 90% of patients resumed normal liver function after stopping use of hepatotoxic drugs [3], elevation of liver enzymes was sometimes accompanied by severe thrombocytopenia and other aggravated side effects [5, 6]. Current chemotherapeutic guidelines require stopping chemotherapy when hepatotoxicity, as indicated by AST and ALT levels, reaches four times the upper normal limit (>100 U l^−1^) [7]. Studies noted that 56–90.5% of cancer patients had to cancel or delay chemotherapy due to serious complications [2, 3]. Changes in chemotherapy courses and drug doses reduced response rates to treatment from 68 to 30% [8].

Use of complementary and alternative medicine (CAM) among patients with cancer has become increasingly popular during the past few decades. A systematic review including 26 surveys of cancer patients from 13 countries showed that the prevalence of CAM use ranged from 7 to 64% [9]. In 2002, a survey of patients with breast, colon or prostate cancer found that 70.2% of respondents used CAM [10]. Traditional Chinese medicine (TCM) is popular among people with a Chinese background.

Taiwan has a two-tiered medical system, comprising both modern medicine and TCM. These two systems operate in parallel and the medical expenses of both are covered by the National Health Insurance system in Taiwan. Studies showed that 9–64% of patients with cancer used TCM in Taiwan [11–16]. According to recent statistics, 72 Western medical hospitals in Taiwan set up departments of TCM, which make it feasible to treat cancer with an integrative system of modern medicine and TCM [17]. While anecdotal evidence shows that TCM protects liver function, TCM used varied across studies and target groups were not confined to cancer patients. Reports of active management of liver injury related to chemotherapy are lacking in the literature [18, 19]. To the best of our knowledge, no study evaluated the effectiveness of TCM using objective indicators among patients with cancer receiving chemotherapy. The purpose of this study was 2-fold: to examine the effectiveness of TCM for protecting the liver from the toxic effects of chemotherapy and to determine if chemotherapy completion rates improved among patients with cancer receiving joint TCM and chemotherapy.

## 2. Methods

We applied a case-control study design. Two senior oncology nurses used a structured questionnaire to independently abstract data from patients' medical records. Inconsistent data were compared, and a consensus on questionable data was reached through discussion among the two data abstractors and the oncologists. Inconsistent data were uncommon and were mostly concerned with determining the timing of the data.

This study was a chart review study and involved no contact with the patients. After data abstraction completed, patient names were replaced by coded numbers to ensure anonymity. The study was approved by the human subjects committee at the study hospital (approval number CRC-08-09-03 issued in May 2009). Written consent was not required, since patient identifiers were not included in the data.

### 2.1. Participants

All patients considered for the study had diagnoses of cancer, were older than 20 years of age, and were treated for cancer using chemotherapy in a teaching hospital in Taipei from January 2004 to December 2004. The study hospital offered both TCM and Western hemo-oncology services. Patients were referred by their oncologist or were self referred for TCM services. Thus, there was no gate-keeping system or formal referral criteria for medical treatment.

A total of 89 patients with cancer were identified from the medical records and their data were analyzed. Of the 89 patients, 37 (42%) ever received TCM prescribed by licensed TCM doctors, while 52 (58%) received modern medical treatments only. Among the 89 patients, 184 chemotherapy courses were identified. Of the 184 courses, 42 (23%) were given in conjunction with TCM (case group), and the remaining 142 (77%) courses without joint TCM served as the control group. Thus, the unit of analysis was one course of chemotherapy.

### 2.2. Treatment Protocols

Patients had blood tests for AST and ALT before the start of a course of chemotherapy and once a week thereafter, according to hospital routine. Patients were advised to fast (nothing by mouth) for eight hours before the blood tests. The blood-testing instrument was calibrated daily by qualified technicians at the study hospital. The TCM medication used were Xiao-Chai-Hu-Tang, Huang-Lian-Jie-Du-Tang or Yin-Chen-Wu-Ling-San during chemotherapy, based on patient conditions as judged by TCM doctors. These TCM medications were traditional multi-component medications. According to TCM theory, these preparations strengthen liver function and detoxify the liver [20, 21] Botanical names for the components of the TCMs are presented in Table 1. TCM medications used in this study were concentrated herbal extracts in the form of powders produced by a GMP-certified pharmaceutical company in Taiwan (Sun Ten Pharmaceutical Co., Ltd, Taichung, Taiwan). The TCM medications were prescribed for three times daily after meal. Each dosage comprised 5 g of TCM powder.

### 2.3. Measurements

A structured four-part questionnaire was used to collect demographic data, including age, gender and disease history, such as hypertension, diabetes mellitus and hepatitis virus status. The second part collected disease and treatment data, including diagnosis, cancer stage, cancer status (newly diagnosed or recurrence), timing of chemotherapy (before or after surgery) and site of radiotherapy. The third part collected chemotherapeutic drugs and chemotherapy course data. Chemotherapeutic drugs which could cause damage to the liver cells of recipients were classified as having potential hepatotoxicity. Even chemotherapeutic drugs which were not classified as having hepatotoxicity could induce poor liver function among patients due to massive cell deaths resulting from chemotherapy and increased metabolic load associated with use of medicine. Therefore, we decided to include all courses of chemotherapy and treat potential hepatotoxicity of the chemotherapeutic drugs as a confounder in this study.

A chemotherapy course was regarded as complete when it was not stopped or postponed, based on recorded chemotherapy regimens. The fourth part collected liver function test data (AST and ALT) 1 week before chemotherapy, during chemotherapy (the last week of chemotherapy), and 2 weeks after chemotherapy.

### 2.4. Data Analysis

The Statistical Package for the Social Sciences (SPSS, version 11.0 for Windows, SPSS Inc, Chicago, IL) and Statistical Analysis System (SAS, version 8.2 SAS Institute, Cary, NC) were used to perform the data analyses. The characteristics of the study participants were summarized as the mean ± standard deviation (SD) for continuous variables and as proportions for categorical variables. Student's *t*-test and chi-square test were used, as appropriate, to analyze group differences. The generalized estimating equations (GEE) model was used to control the effect of study covariates and to analyze the independent effect of TCM therapy on liver function and completion of chemotherapy [22]. The GEE approach has been proposed as a non-parametric and appropriate method to conduct the repeated measurement analysis. This study applied GEE to account for correlated data resulting from repeated measurements across different time points and multiple observations (chemotherapy courses) of the same individual. In all analyses, a 5% significance level was used.

## 3. Results

### 3.1. Subject Demographics

Of the 89 patient records studied, 63% were women, 24% had hypertension, 16% had diabetes mellitus, 8% carried hepatitis B virus and 3% carried hepatitis C virus. The age of the study participants raged from 28 to 88 years (mean 56.03, SD 12.75). Most of the patients had gastrointestinal cancer (40%) and breast cancer (30%). The cancer stages were stage IV (39%), stage III (36%), stage II (18%) and stage I (7%). The majority of patients were newly diagnosed with cancer (83%) (Table 2). Characteristics of the courses of chemotherapy were compared by the case-control status (Table 3). There were no significant differences between the case and control groups except for the cancer diagnosis and cancer stage. More controls had a diagnosis of breast cancer (45.2% versus 14.8%), while more cases had a cancer stage of III and IV (83.8% versus 61.9%). Chemotherapeutic drugs used in this study are listed in the end note to Table 3. About 97% of all chemotherapy courses used chemotherapeutic drugs with potential hepatotoxicity. There were no significant differences between the case and control groups in terms of the use of chemotherapeutic drugs with potential hepatotoxicity.

### 3.2. AST Levels

Among the 184 chemotherapy courses (cases 42, controls 142), the mean AST did not differ significantly between the case and control groups 1 week before and 2 weeks after chemotherapy. However, the case group had significantly lower mean AST values during chemotherapy (24.12 versus 29.20, *P* = .01; Table 4). Median AST values changed little and did not significantly differ between the case and control groups. Median AST values were 23 U l^−1^ for the cases at the three time points; while they were 23, 24 and 24 U l^−1^ for the controls 1 week before, during and 2 weeks after chemotherapy, respectively. The GEE results showed that the case group had significantly decreased AST values during chemotherapy relative to before chemotherapy than did the control group (**β**  = −6.90, *P* = .01). There was no significant difference in AST after chemotherapy relative to before chemotherapy between the case and control groups (**β**  = −3.45, *P* = .30). The main effect of time was significant in that during chemotherapy the participants had higher mean AST values than before chemotherapy (**β**  = 4.42, *P* < .001); AST values before chemotherapy did not differ from those after chemotherapy (**β**  = 1.78, *P* = .11). The main effect of case-control status was not significant (**β**  = 2.48, *P* = .39). After adjusting for study covariates including age, gender, cancer stage, site of radiotherapy, cancer diagnosis and potential hepatotoxicity of the chemotherapeutic drugs, the study results remain quite similar that the significantly lower AST for the case group compared to the control group during chemotherapy was sustained (**β**  = −6.9, *P* = .01). AST values did not differ 2 weeks after chemotherapy between the case and control groups (**β**  = −3.48, *P* = .30). The significant effect of time (higher AST values during chemotherapy than before chemotherapy) was sustained (**β**  = 4.43, *P *< .001) (Table 5).

### 3.3. ALT Levels

Mean ALT did not differ significantly between the case and control groups at all three time points (1 week before, during and 2 weeks after chemotherapy). Nonetheless, the case group had lower mean ALT values during the chemotherapy with borderline significance (22.60 versus 26.33, *P* = .08; Table 4). Median ALT values did not significantly differ between the case and control groups. Median ALT values were 20.5, 22.5 and 21.5 U l^−1^ for the case group, and 18, 19 and 19 U l^−1^ for the controls 1 week before, during and 2 weeks after chemotherapy, respectively. The GEE results showed that the case group had significantly decreased mean ALT values during chemotherapy relative to before chemotherapy than controls (**β**  = −5.99*, P* = .03). There was no significant difference in ALT after chemotherapy relative to before chemotherapy between the case and control groups (**β**  = −0.75, *P* = .83). The main effect of time was significant in that during chemotherapy the participants had higher mean ALT values than before chemotherapy (**β**  = 3.77, *P* = .01); ALT values before chemotherapy did not differ from those after chemotherapy (**β**  = 1.33, *P* = .38). The main effect of case-control status was not significant (**β**  = 0.44, *P* = .84). After adjusting for study covariates including age, sex, cancer stage, site of radiotherapy, cancer diagnosis, and potential hepatotoxicity of the chemotherapeutic drugs, the study results also remain similar that the significantly lower ALT for cases than controls during chemotherapy was sustained (**β**  = −5.95, *P* = .03). ALT values did not differ 2 weeks after chemotherapy between the case and control groups (**β**  = −0.75, *P* = .83). The significant effect of time (higher ALT values during chemotherapy than before chemotherapy) was sustained (**β**  = 3.77, *P* = .01) (Table 6).

### 3.4. Chemotherapy Completion

The chemotherapy completion rate was 76% (32/42) for the case group and 59% (84/142) for the control group (*P* = .04). The GEE results showed that the case group tended to have higher odds of completing chemotherapy, but the results were not statistically significant [odds ratio (OR) = 1.64, *P* = .16]. Furthermore, after adjusting for age, sex, cancer stage, site of radiotherapy, cancer diagnosis, and potential hepatotoxicity of the chemotherapeutic drugs, there were no significant differences between the case and control groups for odds of completing one course of chemotherapy (OR = 1.59, *P* = .21).

## 4. Discussion

We showed that TCM for patients with cancer receiving chemotherapy protected liver function during chemotherapy, as evidenced by lower blood AST and ALT levels, but the effect disappeared 2 weeks after chemotherapy. After adjustment for demographics and disease related factors, TCM lowered AST by 6.9 U l^−1^ (22.9%) and ALT by 5.95 U l^−1^ (19.7%) during chemotherapy. At 2 weeks after chemotherapy, the AST and ALT values returned to levels similar to the value 1 week before chemotherapy for the case group. For the control group, AST and ALT values significantly increased during chemotherapy then decreased to the level only slightly higher than the values before chemotherapy at 2 weeks after chemotherapy. A previous study showed that AST rose during chemotherapy then declined 3 days after chemotherapy [23]. The pattern was consistent with the trend among the controls in this study. The different pattern of AST- and ALT-value changes between the case and control groups in this study suggests efficacy of TCM for liver protection during chemotherapy.

**Figure 1:**
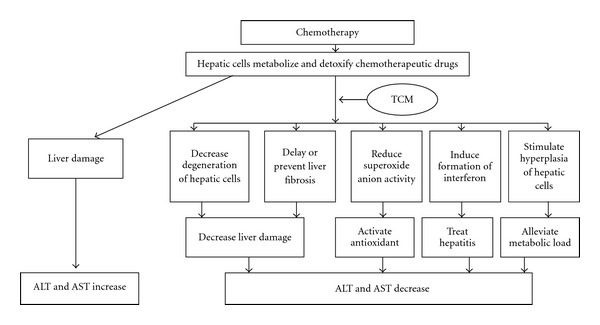
Proposed scheme for the effect of TCMs on liver protection among cancer patients receiving chemotherapy.

The proposed scheme for the effect of TCM on liver protection is presented in Figure 1. During chemotherapy, the drugs stimulate liver and hepatic cells to detoxify and metabolize the chemotherapeutic drugs and this process can cause liver damage such as hepatitis and hepatic necrosis [24, 25]. When the liver is damaged, hepatic cells secrete AST and ALT, which can be detected in the serum. Thus, serum AST and ALT levels were used to assess the extent of damage to hepatic cells. Research shows that TCM decreased liver damage in the case of acute hepatitis through decreasing degeneration of hepatic cells and delaying or preventing liver fibrosis [26, 27]. In addition, TCM has antidotal properties that reduce superoxide anion activity [28]. In other words, TCM could prevent liver injury during chemotherapy through its antioxidant function and decreasing degeneration of liver cells or fibrosis. The other plausible explanations of the beneficial effect of TCM on liver function are hyperplasia of hepatic cells and formation of interferon. TCM preparations such as the rhizome of Chinese goldthread and umbellate pore fungus induce formation of interferon; the rhizome of Chinese goldthread also induces hyperplasia of hepatic cells [29, 30]. Hyperplasia of hepatic cells alleviates metabolic load and interferon treats hepatitis. Therefore, when patients with cancer receive chemotherapy, TCM preparations could stimulate the liver to proliferate and secrete interferon, activating liver self-defenses. At 2 weeks after chemotherapy, when no chemotherapeutic drugs were present to stimulate the hepatic cells, the effect of the TCM preparation disappeared. Determination of the mechanisms linking TCM preparations to improved liver function during chemotherapy were outside the scope of this study. Further studies are needed to examine the underlying mechanisms of TCM action.

While we found that the TCM preparations had no significant effect on completion of chemotherapy among patients with cancer, the patients who had TCM in conjunction with chemotherapy tended toward a higher rate of chemotherapy completion than those who had chemotherapy alone. There were many possible reasons why patients did not complete chemotherapy besides poor liver function and physical health. Reasons for cancellation and delay of chemotherapy were not available for analysis in the current study. More accurate measurement of this variable may yield different results. Further study is needed to examine whether TCM could increase chemotherapy completion rates.

There are a number of TCM preparations that are used to protect or improve liver function based on TCM theory. According to TCM theory, TCM preparations should be adjusted by adding or eliminating ingredients in order to agree with an individual's constitution. Therefore, the contents of TCM preparations vary based on patients' conditions and TCM doctors' recommendations. The TCM preparations used in this study were prescribed by licensed TCM doctors. Three multiple-herb preparations were used as the base, including Huang-Lian-Jie-Du-Tang, Yin-Chen-Wu-ling-san and Xiao-Chai-Hu-Tang. Other studies of TCM preparations showed improved liver function [29, 31–33]. The TCM preparations used in those studies had some overlap of herb components with those used in our current study. Nonetheless, there were still differences in herb components, thus, the comparability of results is limited.

### 4.1. Limitations

This case-control study used course of chemotherapy rather than individual patient as unit of analysis, and GEE was applied to account for the correlated data. This approach could enhance the comparability of the case and control courses and increase data efficiency, since different courses of the same individual could serve either as cases or controls depending on whether TCM was used. Despite these, the allotment of case and control status was based on patient's decisions to seek TCM treatment with or without an oncologist's referral. Therefore, there may be a concern for potential selection bias, but the use of objective rather than subjective indicators as the outcome variables and the use of statistical methods to adjust for confounders may mitigate this concern. This study included patients with different cancer diagnoses and on different chemotherapeutic drugs. Due to the small number of patients in this study, subgroup analysis could not be performed. Nonetheless, the significant effects of lowered AST and ALT were sustained given that the GEE approach was used to account for correlated observations and adjustment for potential confounders in the study. A future study with more-homogeneous samples and a larger sample size is needed. Potential interactions between TCMs and chemotherapeutic drugs should be examined in future studies. There is a lack of information on other indicators for liver function such as GGT, bilirubin and ALD in this study. This study only examined a limited spectrum of patient outcomes; in addition to a more-complete liver profile, information about the treatment efficacy such as tumor reduction, relapse and blood profile should be considered in future studies.

## 5. Conclusion

Integrative care for cancer patients using modern medicine and TCM is an emerging trend. We demonstrated that use of TCM preparations improved liver function during chemotherapy among our patients with cancer receiving chemotherapy. Randomized controlled trails are warranted in order to confirm the effectiveness of various TCM preparations for protection of liver function among cancer patients receiving chemotherapy.

## Figures and Tables

**Table 1:** Botanical names for the components of TCMs.

TCM medication		
Xiao-Chai-Hu-Tang	Huang-Lian-Jie-Du-Tang	Yin-Chen-Wu-Ling-San
Buplerum chinense DC.	The rhizome of Chinese goldthread	Poria cocos tuckahoe
Pinellia ternate	Scutellaria baicalensis Georgi	Agaric umbellate pore fungus
Ginseng	Phellodendri Cortex	Largehead atractylodes Rh
Scutellaria baicalensis Georgi	Gardeniae Fructus	Cassia twigs, ramulus cinnamomi
Glycyrrhizae Radix		Alismatis rhizoma
Ginger		Artemisiae scopariae herba
Big jujube		

**Table 2:** Characteristics of the study participants (*N* = 89).

	*n*	Percentage (%)
Age (years)		
28–40	8	9.0
41–50	26	29.2
51–60	23	25.8
61–70	20	22.5
71–80	10	12.4
80–88	2	1.1
Sex		
Male	33	37.1
Female	56	62.9
Cancer diagnosis		
Breast cancer	27	30.3
Gastrointestinal cancer	36	40.4
Lung cancer	7	7.9
Lip, oral cavity, pharynx and throat cancer	5	5.6
Genital and urological cancer	4	4.5
Lymph and blood system cancer	7	7.9
Bone and connective tissue cancer	2	2.2
Cancer stages		
I	6	6.7
II	16	18.0
III	32	36.0
IV	35	39.3
Cancer status		
Newly diagnosed	74	83.1
Relapse	15	16.9
Hepatitis virus carrier		
B	7	7.9
C	3	3.4

**Table 3:** Characteristics by the case-control status (*N* = 184).

	Case (*n* = 42)	Control (*n* = 142)	*t*/*χ* ^2^	*P*
Age	Mean = 58.43; SD = 12.28	Mean = 55.17; SD = 10.12	1.57	.12
Sex			2.28	.09
Male	14 (33.3%)	66 (46.5%)		
Female	28 (66.7%)	76 (53.5%)		
Cancer diagnosis			43.47	<.0001
Breast cancer	21 (14.8%)	19 (45.2%)		
Gastrointestinal cancer	88 (62.0%)	8 (19.0%)		
Lung cancer	15 (10.6%)	2 (4.8%)		
Lip, oral cavity, pharynx and throat cancer	2 (1.4%)	4 (9.5%)		
Genital and urological cancer	7 (4.9%)	3 (7.1%)		
Lymph and blood system cancer	9 (6.3%)	3 (7.1%)		
Bone and connective tissue cancer	0 (0.0%)	1 (2.4%)		
Others	0 (0.0%)	2 (4.8%)		
Cancer stages			19.15	<.001
I	2 (1.4%)	7 (16.7%)		
II	21 (14.8%)	9 (21.4%)		
III	51 (35.9%)	14 (33.3%)		
IV	68 (47.9%)	12 (28.6%)		
Site of radiotherapy			5.11	.08
No	58 (40.8%)	23 (54.8%)		
Abdomen	45 (31.7%)	6 (14.3%)		
Non-abdomen	39 (27.5%)	13 (31.0%)		
Hepatotoxicity of the chemotherapeutic drugs			0.13	.72
Yes	41 (97.6%)	137 (96.5%)		
No	1 (2.4%)	5 (3.5%)		

*t* and *χ* ^2^ are respective statistical values from Student's; *t*-test and chi-squared test; the *P-*value is the probability assuming that null hypothesis is true (two-tailed test); the significance level was set to *P* < .05. The chemotherapeutic drugs used in this study were Ara-C, bleomycin, carboplatin, cisplatin, cyclophosphamide, dacarbazine, doxorubicin, etoposide, fluorouracil, formoxol, gemcitabine, ifosfamide, mitoxantrone, methotrexate, mitomycin C, oxaliplatin, taxol and taxotere (with potential hepatotoxicity); arimidex, CPT-11, faslodex, herceptin, navelbine, tamoxifen, UFUR and vinblastine (without hepatotoxicity).

**Table 4:** Mean AST and ALT values by case-control status.

	Case (*n* = 42)		Control (*n* = 142)		*t*	*P*
	Mean	SD	Mean	SD		
AST before chemotherapy	26.55	16.04	24.76	10.09	−0.82	.41
AST during chemotherapy	24.12	5.37	29.20	20.07	2.61	.01
AST after chemotherapy	25.49	7.73	26.67	14.32	0.50	.61
ALT before chemotherapy	24.17	15.34	22.70	15.65	−0.53	.59
ALT during chemotherapy	22.60	6.97	26.33	20.90	1.76	.08
ALT after chemotherapy	24.74	15.88	24.35	17.08	−0.13	.89

*t* is the statistical value from Student's; *t*-test; the *P*-value is the probability assuming that the null hypothesis is true (two-tailed test); the significance level was set to *P* < .05.

**Table 5:** Crude and net effect of TCM use on AST.

	Estimate	95% CI		Z	*P*
Crude effect					
TCM cases	2.48	−3.21	8.19	0.85	.39
During chemotherapy	4.42	2.01	6.82	3.60	<.001
After chemotherapy	1.78	−0.46	4.04	1.56	.11
Case × During	−6.90	−12.30	−1.50	−2.51	.01
Case × After	−3.45	−10.08	3.17	−1.02	.30
Net effect					
TCM cases	3.61	−2.47	9.70	1.16	.24
During chemotherapy	4.43	2.01	6.85	3.59	.0003
After chemotherapy	1.81	−0.44	4.05	1.60	.11
Case × During	−6.9	−12.30	−1.50	−2.51	.01
Case × After	−3.48	−10.08	3.11	−1.03	.30

The reference groups were controls for case–control status and before chemotherapy for time.

Crude effect was unadjusted results. Net effect was results adjusted for age, sex, cancer stage, site of radiotherapy, cancer diagnosis and potential hepatotoxicity of the chemotherapeutic drugs.

*Z* is the statistical value from GEE; the *P* value is the probability assuming that the null hypothesis is true (two-tailed test); the significance level was set to *P* < .05; “×" indicates an interaction.

**Table 6:** Crude and net effect of TCM use on ALT.

	Estimate	95% CI		Z	*P*
Crude effect					
TCM cases	0.44	−3.99	4.88	0.20	0.84
During chemotherapy	3.77	0.68	6.86	2.39	0.01
After chemotherapy	1.33	−1.68	4.34	0.87	0.38
Case × During	−5.99	−11.50	−0.47	−2.13	0.03
Case × After	−0.75	−7.81	6.29	−0.21	0.83
Net effect					
TCM cases	−0.33	−5.47	4.81	−0.13	0.89
During chemotherapy	3.77	0.68	6.87	2.39	0.01
After chemotherapy	1.33	−1.68	4.34	0.87	0.38
Case × During	−5.95	−11.47	−0.44	−2.12	0.03
Case × After	−0.75	−7.79	−6.27	−0.21	0.83

The reference groups were controls for case-control status and before chemotherapy for time.

Crude effect was unadjusted results. Net effect was results adjusted for age, sex, cancer stage, site of radiotherapy, cancer diagnosis, and potential hepatotoxicity of the chemotherapeutic drugs.

*Z* is the statistical value from the GEE; the *P* value is the probability assuming that the null hypothesis is true (two-tailed test); the significance level was set to *P* < .05; “ ×" indicates an interaction.

## References

[1] Floyd J, Mirza I, Sachs B, Perry MC (2006). Hepatotoxicity of chemotherapy. *Seminars in Oncology*.

[2] Hurria A, Leung D, Trainor K, Borgen P, Norton L, Hudis C (2003). Factors influencing treatment patterns of breast cancer patients age 75 and older. *Critical Reviews in Oncology/Hematology*.

[3] Law CC, Fu YT (2002). Postoperative adjuvant 5-fluororuacil plus levamisole chemotherapy for stage III colon carcinoma: 7-year experience in a single institution. *Journal of Hong Kong College of Radiologists*.

[4] Alvarado Y, Tsimberidou A, Kantarjian H (2003). Pilot study of Mylotarg, idarubicin and cytarabine combination regimen in patients with primary resistant or relapsed acute myeloid leukemia. *Cancer Chemotherapy and Pharmacology*.

[5] Mericer C, Ciccilini J, Pourroy B (2006). Dose individualized of carboplatin after a 120-hour infusion schedule: higher dose intensity but fewer toxicities. *Therapeutic Drug Monitoring*.

[6] Xie H, Griskevicius L, Ståhle L (2006). Pharmacogenetics of cyclophosphamide in patients with hematological malignancies. *European Journal of Pharmaceutical Sciences*.

[7] Tsai HC, Liaw YS, Chen YJ, Gau CS, Yu CJ, Luh KT (2004). Management of anti-tuberculosis drug-related hepatotoxicity: comparison of the fluoroquinolone-containing regimen and re-challenge with the standard regimen. *Thoracic Medicine*.

[8] D’hondt R, Paridaens R, Wildiers H (2004). Safety and efficacy of weekly docetaxel in frail and/or elderly patients with metastatic breast cancer: a phase II study. *Anti-Cancer Drugs*.

[9] Ernst E, Cassileth BR (1998). The prevalence of complementary/alternative medicine in cancer: a systematic review. *Cancer*.

[10] Patterson RE, Neuhouser ML, Hedderson MM (2002). Types of alternative medicine used by patients with breast, colon, or prostate cancer: predictors, motives, and costs. *Journal of Alternative and Complementary Medicine*.

[11] Liu JM, Chu HC, Chin YH (1997). Cross sectional study of use of alternative medicines in Chinese cancer patients. *Japanese Journal of Clinical Oncology*.

[12] Tseng YH, Lin TH, Hung CA (2005). Use of complementary therapies in community-dwelling adults in Taichung area. *The Chung Shan Medical Journal*.

[13] Shi HZ, Chiu HC, Ker CG, Chen YF, Huang YS (2005). Utilization of medical resources by cancer patients from the Chi-Ching District in Kaohsiung. *Formosa Journal of Healthcare Administration*.

[14] Hou YC, Ting HH, Tseng YC, Hsieh YH, Huang CC (2006). Study of application of alternative medicine in cancer patients-survey of a regional hospital surgery outpatient department. *Journal of Chinese Medicine*.

[15] Ting HH, Huang CC, Chiu HL, Wu CT, Chou CC, Hou YC (2007). Utilizations of complementary and alternative medicine in breast cancer patients—survey of a regional hospital surgery outpatient department. *Chinese Journal of Integrated Traditional and Western Medicine*.

[16] Yang C, Chien L-Y, Tai C-J (2008). Use of complementary and alternative medicine among patients with cancer receiving outpatient chemotherapy in Taiwan. *Journal of Alternative and Complementary Medicine*.

[17] Department of Health, Taiwan. Evaluation of the Department of Chinese Medicine. http://www.doh.gov.tw/CHT2006/DM/SEARCH_MAIN.aspx?keyword=%u4e2d%u91ab%u91ab%u9662.

[18] Tostmann A, Boeree MJ, Aarnoutse RE, de Lange WCM, van der Ven AJAM, Dekhuijzen R (2008). Antituberculosis drug-induced hepatotoxicity: concise up-to-date review. *Journal of Gastroenterology and Hepatology*.

[19] Núñez M (2006). Hepatotoxicity of antiretrovirals: incidence, mechanisms and management. *Journal of Hepatology*.

[20] Zhang ZJ *On Cold Damage (Shang Han Lun)*.

[21] Zhang ZJ *Synopsis of Golden damage (Jin Gui Yao Lue)*.

[22] Zeger SL, Liang KY (1986). Longitudinal data analysis for discrete and continuous outcomes. *Biometrics*.

[23] Babaoglu MO, Karadag O, Saikawa Y (2004). Hepatotoxicity due to a possible interaction between cytosine arabinoside and dipyridamole: a case report. *European Journal of Clinical Pharmacology*.

[24] Jaeschke H, Gores GJ, Cederbaum AI, Hinson JA, Pessayre D, Lemasters JJ (2002). Mechanisms of hepatotoxicity. *Toxicological Sciences*.

[25] McDonald GB, Tirumali N (1984). Intestinal and liver toxicity of antineoplastic drugs. *Western Journal of Medicine*.

[26] Chen MH, Gao ZQ (2003). Treatment of hepatic encephalopathy and liver cirrhosis with Chinese medicine: a case report. *Taiwan Journal of Clinical Chinese Medicine*.

[27] Lee JR, Park SJ, Lee H-S (2009). Hepatoprotective activity of licorice water extract against Cadmium-induced toxicity in rats. *Evidence-Based Complementary and Alternative Medicine*.

[28] Liao H, Banbury LK, Leach DN (2008). Antioxidant activity of 45 Chinese herbs and the relationship with their TCM characteristics. *Evidence-Based Complementary and Alternative Medicine*.

[29] Cheng CH (2005). Experiences of treating viral hepatitis B with traditional Chinese medicine. *Journal of Traditional Chinese Internal Medicine*.

[30] Chow KC, Chen KY (1994). The viewpoints of modern medicine and Chinese medicine toward the diagnosis and treatment of cancers. *Therapeutic Radiology Oncology*.

[31] Zou Y, Yang Y, Li J, Li W, Wu Q (2006). Prevention of hepatic injury by a traditional Chinese formulation, BJ-JN, in mice treated with Bacille-Calmette-Guérin and lipopolysaccharide. *Journal of Ethnopharmacology*.

[32] Lin T-H, Ng L-T, Yen F-L, Lin C-C (2007). Hepatoprotective effects of Chai-Hu-Ching-Kan-Tang on acetaminophen-induced acute liver injury in rats. *American Journal of Chinese Medicine*.

[33] Tseng S-H, Chien T-Y, Tzeng C-F, Lin Y-H, Wu C-H, Wang C-C (2007). Prevention of hepatic oxidative injury by Xiao-Chen-Chi-Tang in mice. *Journal of Ethnopharmacology*.

